# Usefulness of N-terminal pro-B-type natriuretic peptide in patients admitted to the intensive care unit: a multicenter prospective observational study

**DOI:** 10.1186/1471-2253-14-16

**Published:** 2014-03-10

**Authors:** Chin Kook Rhee, So Yeon Lim, Shin Ok Koh, Won-Il Choi, Young-Joo Lee, Gyu Rak Chon, Je Hyeong Kim, Jae Yeol Kim, Jaemin Lim, Sunghoon Park, Ho Cheol Kim, Jin Hwa Lee, Ji Hyun Lee, Jisook Park, Younsuck Koh, Gee Young Suh, Seok Chan Kim

**Affiliations:** 1 Division of Pulmonary and Critical Care Medicine, Department of Medicine, Seoul St. Mary’s Hospital, The Catholic University of Korea, 505 Banpo-Dong, Seoul, Seocho-Gu 137-701, South Korea; 2 Division of Pulmonary and Critical Care Medicine, Department of Medicine, Samsung Medical Center, Sungkyunkwan University School of Medicine, Seoul, South Korea; 3 Division of Critical Care Medicine, Department of Anesthesiology and Pain Medicine, Severance Hospital, and Anesthesia and Pain Research Institute, Yonsei University College of Medicine, Seoul, South Korea; 4 Division of Pulmonary and Critical Care Medicine, Department of Medicine, Keimyung University, Dongsan Hospital, Daegu, South Korea; 5 Department of anesthesiology, Aju university college of medicine, Suwon, South Korea; 6 Division of Pulmonary and Critical Care Medicine, Department of Medicine, Chungju hospital, School of medicine of Konkuk University, Chungju, South Korea; 7 Sleep and Critical Care Medicine, Department of Medicine, Korea University Ansan Hospital, Ansan, South Korea; 8 Division of Pulmonary and Critical Care Medicine, Department of Medicine, Chung-Ang University College of Medicine, Seoul, South Korea; 9 Division of Pulmonary and Critical Care Medicine, Department of Medicine, Gangneung Asan Hospital, Gangneung, University of Ulsan Medical College of internal medicine, Gangneung, South Korea; 10 Department of Pulmonary, Allergy and Critical Care Medicine, Hallym University Sacred Heart Hospital, Ahnyang, South Korea; 11 Division of Pulmonary and Critical Care Medicine, Department of Medicine, College of Medicine, Gyeongsang Institute of Health Sciences, Gyeongsang National University, Jinju, South Korea; 12 Division of Pulmonary and Critical Care Medicine, Department of Medicine, Ewha Womans University School of Medicine, Seoul, South Korea; 13 Division of Pulmonary and Critical Care Medicine, Department of Medicine, Bundang CHA hospital CHA University, Bundang, South Korea; 14 Department of Multimedia, Seoul Women’s University, Seoul, South Korea; 15 Division of Pulmonary and Critical Care Medicine, Department of Medicine, Asan Medical Center, University of Ulsan College of Medicine, Seoul, South Korea

**Keywords:** N-terminal pro-B-type natriuretic peptide, Intensive care unit, Critical care, Prognosis

## Abstract

**Background:**

The role of N-terminal pro-B-type natriuretic peptide (NT-pro-BNP) as a prognostic factor in patients admitted to the intensive care unit (ICU) is not yet fully established. We aimed to determine whether NT-pro-BNP is predictive of ICU mortality in a multicenter cohort of critically ill patients.

**Methods:**

A total of 1440 patients admitted to 22 ICUs (medical, 14; surgical, six; multidisciplinary, two) in 15 tertiary or university-affiliated hospitals between July 2010 and January 2011 were assessed. Patient data, including NT-pro-BNP levels and Simplified Acute Physiology Score (SAPS) 3 scores, were recorded prospectively in a web-based database.

**Results:**

The median age was 64 years (range, 53–73 years), and 906 (62.9%) patients were male. The median NT-pro-BNP level was 341 pg/mL (104–1,637 pg/mL), and the median SAPS 3 score was 57 (range, 47–69). The ICU mortality rate was 18.9%, and hospital mortality was 24.5%. Hospital survivors showed significantly lower NT-pro-BNP values than nonsurvivors (245 pg/mL [range, 82–1,053 pg/mL] *vs*. 875 pg/mL [241–5,000 pg/mL], respectively; *p* < 0.001). In prediction of hospital mortality, the area under the curve (AUC) for NT-pro-BNP was 0.67 (95% confidence interval [CI], 0.64–0.70) and SAPS 3 score was 0.83 (95% CI, 0.81–0.85). AUC increment by adding NT-pro-BNP is minimal and likely no different to SAPS 3 alone.

**Conclusions:**

The NT-pro-BNP level was more elevated in nonsurvivors in a multicenter cohort of critically ill patients. However, there was little additional prognostic power when adding NT-pro-BNP to SAPS 3 score.

## Background

B-type natriuretic peptide (BNP) is released from cardiac ventricles in response to increased wall tension [1]. Measurement of BNP is useful in establishing the diagnosis of heart failure [2]. N-terminal pro-B-type natriuretic peptide (NT-pro-BNP), a precursor of BNP, provides prognostic information superior to that obtained from BNP in patients with myocardial infarction [3]. NT-pro-BNP has been shown to be a good prognostic marker in patients with cardiac disease [4].

NT-pro-BNP levels are elevated not only in patients with cardiac disease but also in critically ill patients. Okkonen *et al*. [5] showed that the NT-pro-BNP level on patient admission is commonly elevated in patients with acute respiratory failure. Wang *et al*. [6] reported that elevated NT-pro-BNP levels may prove to be a powerful predictor of mortality in septic patients. NT-pro-BNP level may be a prognostic marker in critically ill patients [7-10]. However, few large-scale multicenter studies have assessed NT-pro-BNP as a prognostic factor. Also, little is known regarding the correlation between NT-pro-BNP and clinical parameters in critically ill patients. Moreover, it is not clear in which conditions NT-pro-BNP is elevated. Thus, by using a large cohort we aimed to assess the prognostic value of NT-pro-BNP level in patients admitted to the intensive care unit (ICU) and analyzed the relationship between the level of NT-pro-BNP and clinical parameters.

## Methods

We used data from the “Validation of simplified acute physiology score 3 in Korean ICUs” (VSKI) study cohort. VSKI was a prospective multicenter cohort study that aimed to validate the simplified acute physiology score (SAPS) 3 in Korean ICU patients that was performed by the Korean Study group on Respiratory Failure (KOSREF) between July 1st, 2010 and January 31st, 2011. VSKI included 22 ICUs (medical, 14; surgical, six; multidisciplinary, two) in 15 tertiary or university-affiliated hospitals. The study was approved by the institutional review board of the Seoul St. Mary’s Hospital, Samsung Medical Center, Severance Hospital, Keimyung University Dongsan Hospital, Aju University Hospital, Konkuk University Chungju Hospital, Korea University Ansan Hospital, Armed Forces Capital Hospital, Chung-Ang University Hospital, Gangneung Asan Hospital, Hallym University Sacred Heart Hospital, Gyeongsang National University Hospital, Ewha Womans University Hospital, Bundang CHA Hospital and Asan Medical Center. The requirement for informed consent was waived because of the observational nature of the study.

### Patients

All patients admitted to the 22 ICUs during the study period were eligible for the present study. Patients whose NT-pro-BNP levels were measured on ICU admission were included in the study. We excluded patients who were younger than 17 years. Patients who were transferred from other participating ICUs were also excluded. For patients with two or more admissions to the ICU during the same hospital stay, only the data from the first admission were used. Patients whose ICU or hospital mortality was uncertain were excluded in the analysis.

### Data collection

Patient data were recorded prospectively in a web-based database. We obtained data on demographic characteristics (age, gender, body weight, and height), underlying disease, SAPS 3, sequential organ failure assessment (SOFA) score, severe sepsis, or septic shock on ICU admission, acute lung injury (ALI) or acute respiratory distress syndrome (ARDS) on ICU admission, admission category (medical or surgical), admission diagnosis, organ support (mechanical ventilation, renal replacement therapy, and use of vasopressors), length of ICU stay and hospital stay, and mortality on ICU and hospital discharge. The medical history of each patient was reviewed thoroughly, and the initial vital signs on ICU admission were recorded. Laboratory data, including complete blood cell count, chemistry, and arterial blood gas analysis were collected within 24 h of ICU admission. Illness severity was assessed using the SAPS 3 score. Blood NT-pro-BNP level was determined together with all SAPS 3 variables.

### Data analysis

We compared the level of NT-pro-BNP on admission in patients with hospital survivors and nonsurvivors. Then, we compared the mortality rate according to the quintile of NT-pro-BNP. We also compared the level of NT-pro-BNP according to the reason of ICU admission and analyzed correlation between NT-pro-BNP and clinical parameters. We calculated prognostic power of NT-pro-BNP to predict mortality and compared with that of SAPS 3 score. Finally, we analyzed if there was additive benefit when combining NT-pro-BNP with SAPS 3 score.

### Statistical analysis

Because most of the data were not normally distributed, all results are presented as medians and interquartile ranges (IQRs), or as numbers (percentages) of patients. Differences between groups were assessed using chi-squared test or Fisher’s exact test for categorical variables, as appropriate. For continuous variables, differences between two groups were assessed by the Mann–Whitney *U*-test and differences among four groups were assessed by the Kruskal–Wallis test. Correlations between continuous variables were assessed by Spearman’s test. Receiver operating characteristic (ROC) curves were generated for NT-pro-BNP and SAPS 3. SAPS 3 and NT-pro-BNP were evaluated for their association with hospital survival by logistic regression analysis. NT-pro-BNP was transformed into a natural log (ln) scale because of the wide range of levels. All tests were two sided, and *P* values less than 0.05 were considered to indicate statistical significance. All statistical analyses were performed using PASW Statistics, version 17 (SPSS, Chicago, IL, USA).

## Results

**Figure 1:**
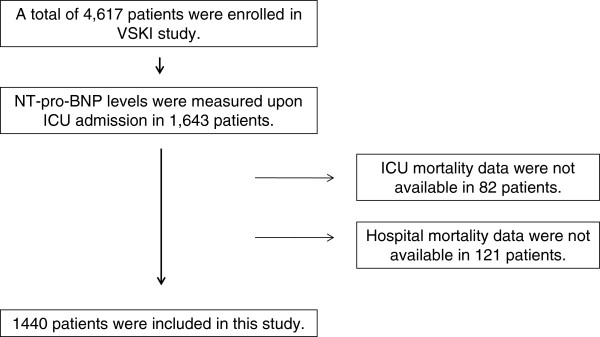
Enrolled patients.

NT-pro-BNP levels in 1,643 patients were measured upon ICU admission. Among them, ICU mortality data were available for 1,561 patients, and hospital mortality data were available in 1440 patients. Thus, 1440 patients were included in the study (Figure 1). The median age was 64 years (range, 53–73 years), and 906 (62.9%) were male. On ICU admission, 309 (21.5%) patients had severe sepsis or septic shock, and 123 (8.5%) were ALI or ARDS. The median NT-pro-BNP was 341 pg/mL (range, 104–1,637 pg/mL), and the median SAPS 3 score was 57 (range, 47–69). The ICU mortality rate was 18.9%, and the hospital mortality rate was 24.5%. The baseline characteristics of the patients are shown in Table 1.

**Table 1:** Baseline characteristics of patients (n = 1440)

**Characteristics**	**Median (IQR) or No. (%)**
Age in years (range)	64 (53–73)
Male	906 (62.9)
Comorbidities	
Cirrhosis	157 (10.9)
Hypertension	544 (37.8)
IHD	160 (11.1)
CHF	108 (7.5)
DM	363 (25.2)
CRF	144 (10.0)
Cancer	544 (37.8)
Status at ICU admission	
Severe sepsis or septic shock	309 (21.5)
ALI or ARDS	123 (8.5)
Admission category	
Medical	835 (58.0)
Surgical	605 (42.0)
Reason for ICU admission	
Basic & observational^**^	583 (40.5)
Cardiovascular	264 (18.3)
Digestive	62 (4.3)
Hepatic failure	86 (6.0)
Neurologic	35 (2.4)
Renal	21 (1.5)
Respiratory	310 (21.5)
NT-pro-BNP (pg/mL)	341 (104–1,637)
SAPS 3 score	57 (47–69)
SOFA score	7 (3–11)
ICU day	7 (4–16)
ICU mortality	272 (18.9)
Hospital day	18 (10–35)
Hospital mortality	353 (24.5)

^**^Basic and observational intensive care were defined as management of a patient in the ICU for surveillance, simple weaning from a ventilator after surgery, routine post-surgery care, or needing complex nursing care or monitoring of drug intoxication without organ dysfunction.

IQR = interquartile range; IHD = ischemic heart disease; CHF = congestive heart failure; DM = diabetes mellitus; CRF = chronic renal failure; ICU = intensive care unit; ALI = acute lung injury; ARDS = acute respiratory distress syndrome; SAPS 3 = Simplified Acute Physiology Score (SAPS) 3.

**Figure 2:**
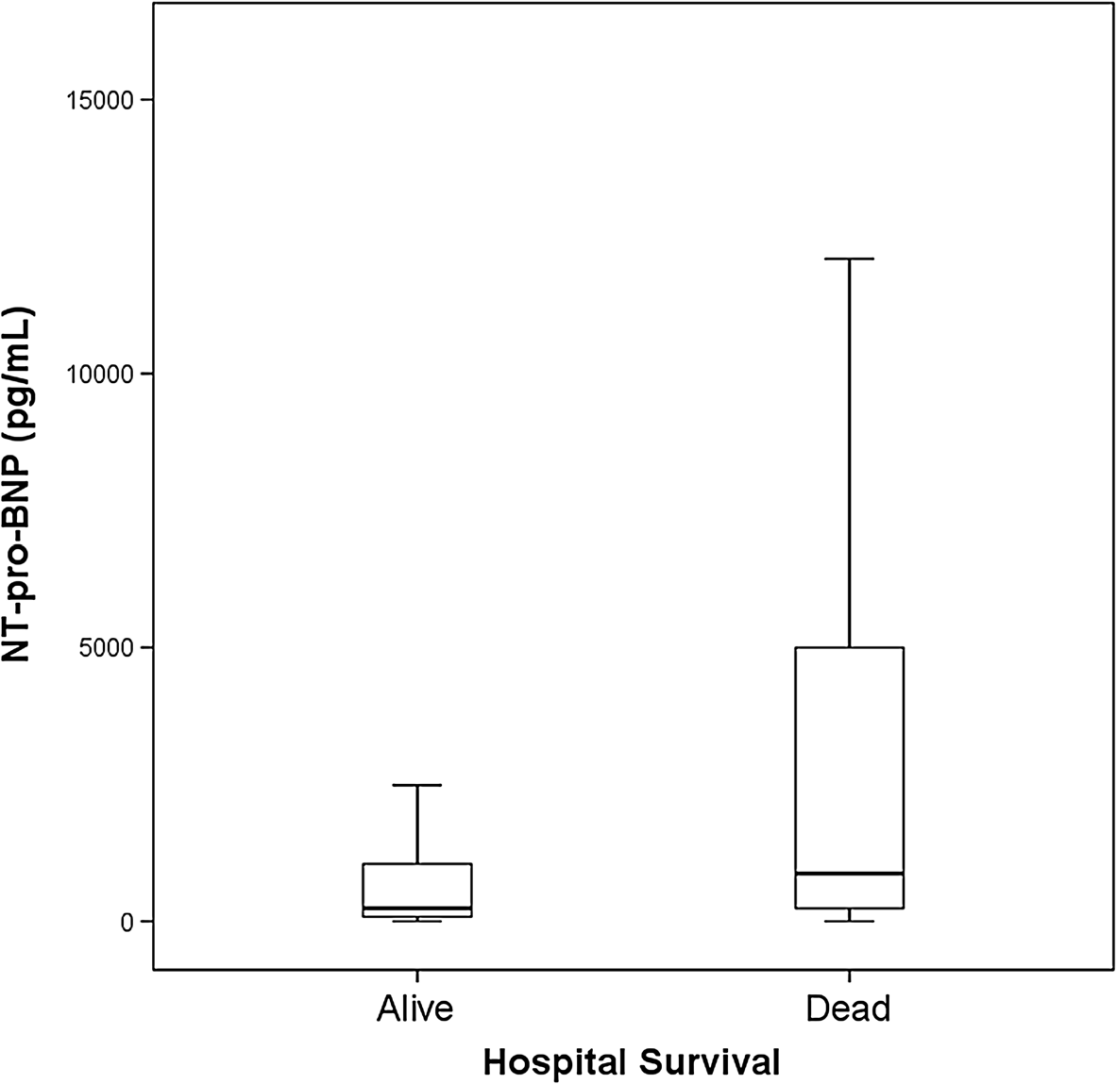
**Box and whisker plots for hospital survival.** The NT-pro-BNP level was significantly higher in nonsurvivors than survivors.

ICU survivors had significantly lower NT-pro-BNP values than ICU nonsurvivors (268 pg/mL [range, 80–1,140 pg/mL] *vs*. 1,021 pg/mL [range, 232–6,256 pg/mL], respectively; *p* < 0.001). Likewise, hospital survivors were characterized by significantly lower NT-pro-BNP values than hospital nonsurvivors (245 pg/mL [range, 82–1,053 pg/mL] *vs*. 875 pg/mL [range, 241–5,000 pg/mL], respectively; *p* < 0.001). Figure 2 shows box and whisker plots for hospital survival.

**Figure 3:**
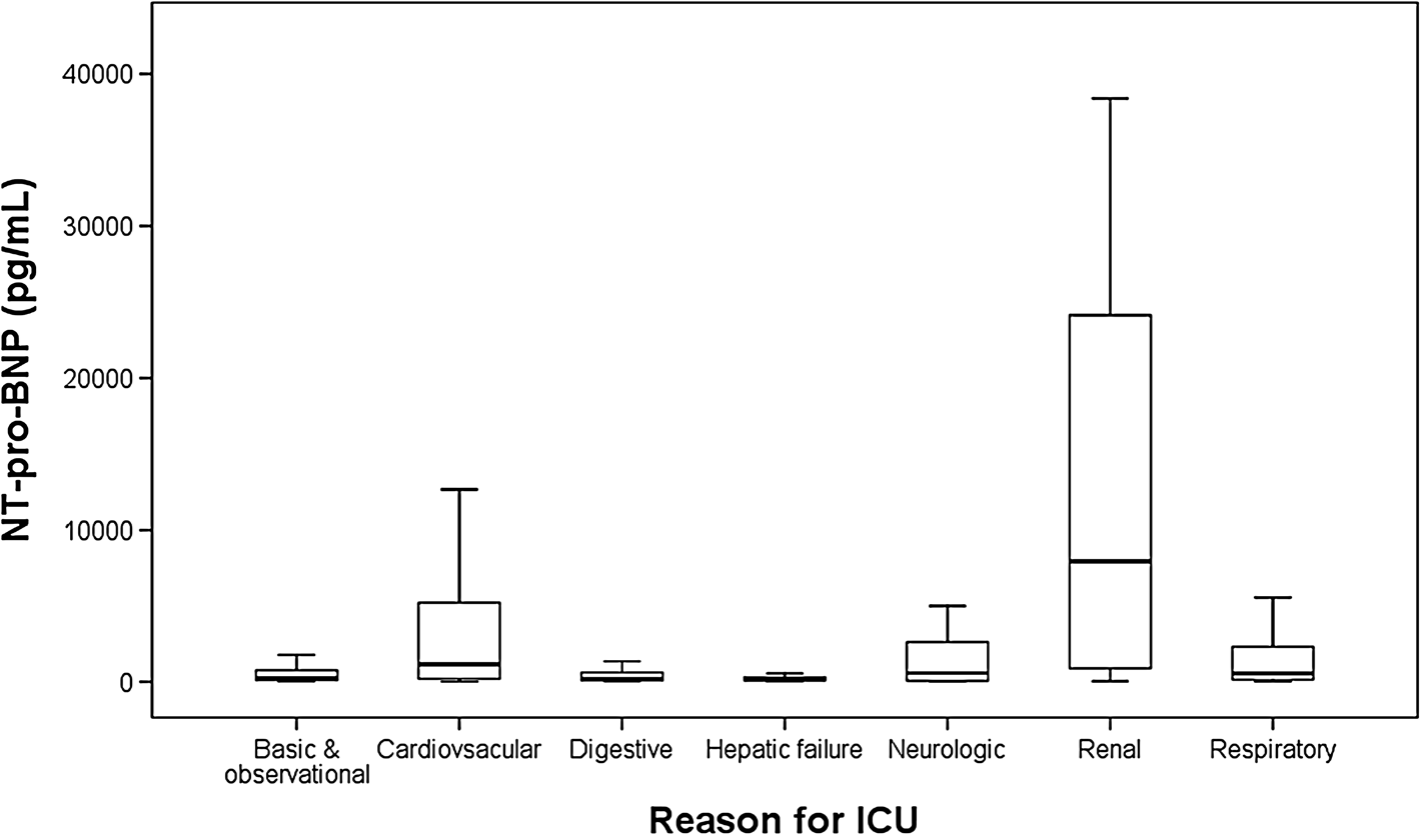
**Box and whisker plots for the reason for ICU admission.** NT-pro-BNP levels varied according to the reason for ICU admission.

The NT-pro-BNP level was significantly different according to the reason for ICU admission (*p* < 0.001; Figure 3). The level of NT-pro-BNP was highest in patients whose reason for ICU admission was a renal complication (7,946 pg/mL [range, 890–24,118 pg/mL]), followed by a cardiovascular complication (1,154 pg/mL [range, 190–5,217 pg/mL]). The level of NT-pro-BNP was significantly different according to underlying disease or clinical situation (Table 2). Significant correlations between NT-pro-BNP level and many clinical parameters were found (Table 3).

**Table 2:** Level of NT-pro-BNP (pg/mL) according to the underlying disease or clinical situation

**Variables**	**No. (%)**	**Median (IQR)**	***p*-value**
Female	534 (37.1%)	420 (137–1,780)	0.008
Male	906 (62.9%)	292 (84–1,520)	
Cirrhosis (-)	1,283 (89.1%)	385 (109–1,808)	<0.001
Cirrhosis (+)	157 (10.9%)	187 (83–526)	
Hypertension (-)	896 (62.2%)	276 (86–1,208)	<0.001
Hypertension (+)	544 (37.8%)	456 (125–2,384)	
IHD (-)	1,280 (88.9%)	296 (93–1,309)	<0.001
IHD (+)	160 (11.1%)	967 (251–4,894)	
CHF (-)	1,332 (92.5%)	299 (93–1,382)	<0.001
CHF (+)	108 (7.5%)	1,226 (413–8,346)	
DM (-)	1,077 (74.8%)	284 (94–1,223)	<0.001
DM (+)	363 (25.2%)	611 (129–3,373)	
CRF (-)	1,296 (90.0%)	276 (91–1,127)	<0.001
CRF (+)	144 (10.0%)	2,799 (773–16,768)	
Cancer (-)	896 (62.2%)	390 (101–2,214)	0.027
Cancer (+)	544 (37.8%)	285 (108–1,024)	
Severe sepsis or septic shock (-)	1,131 (78.5%)	269 (85–1,112)	<0.001
Severe sepsis or septic shock (+)	309 (21.5%)	890 (206–7,946)	
ALI or ARDS (-)	1,317 (91.5%)	309 (100–1,389)	<0.001
ALI or ARDS (+)	123 (8.5%)	827 (191–3,504)	
Elective surgery	502 (34.9%)	195 (78–492)	<0.001
Emergency surgery	103 (7.2%)	233 (97–981)	
Surgery (-)	835 (58.0%)	604 (148–3,170)	
MV (-)	770 (53.5%)	264 (83–1,064)	<0.001
MV (+)	670 (46.5%)	456 (129–2,542)	
Vasoactive drug (-)	904 (62.8%)	237 (79–828)	<0.001
Vasoactive drug (+)	536 (37.2%)	721 (178–3,720)	
RRT (-)	1,268 (88.1%)	278 (93 – 1,164)	<0.001
RRT (+)	172 (11.9%)	2,377 (413–18,751)	

IQR = interquartile range; IHD = ischemic heart disease; CHF = congestive heart failure; DM = diabetes mellitus; CRF = chronic renal failure; ALI = acute lung injury; ARDS = acute respiratory distress syndrome; MV = mechanical ventilation; RRT = renal replacement therapy.

**Table 3:** Correlations between NT-pro-BNP and clinical parameters

**Clinical parameters**	**Correlation coefficient (p)**	***p*-value**
Age	0.19	<0.001
Lowest MAP	-0.22	<0.001
Highest HR	0.25	<0.001
Highest BT	0.13	<0.001
Lowest platelet	-0.11	<0.001
Highest total bilirubin	-0.07	0.006
Highest BUN	0.40	<0.001
Highest Cr	0.33	<0.001
Lowest Na	-0.12	<0.001
Highest K	0.14	<0.001
Lowest pH	-0.09	0.001
Lowest HCO_3_^-^	-0.17	<0.001
Lowest PaO_2_	-0.25	<0.001
Highest PaCO_2_	-0.06	0.029
Highest GCS	-0.10	<0.001
Lowest PF ratio	-0.26	<0.001
Input	0.07	0.013
Urine output	-0.11	<0.001

MAP = mean arterial pressure, HR = heart rate, BT = body temperature, BUN = blood urea nitrogen, Cr = creatinine, Na = sodium, K = potassium, GCS = Glasgow Coma Scale, PF ratio = PaO_2_/FIO_2_ ratio.

**Figure 4:**
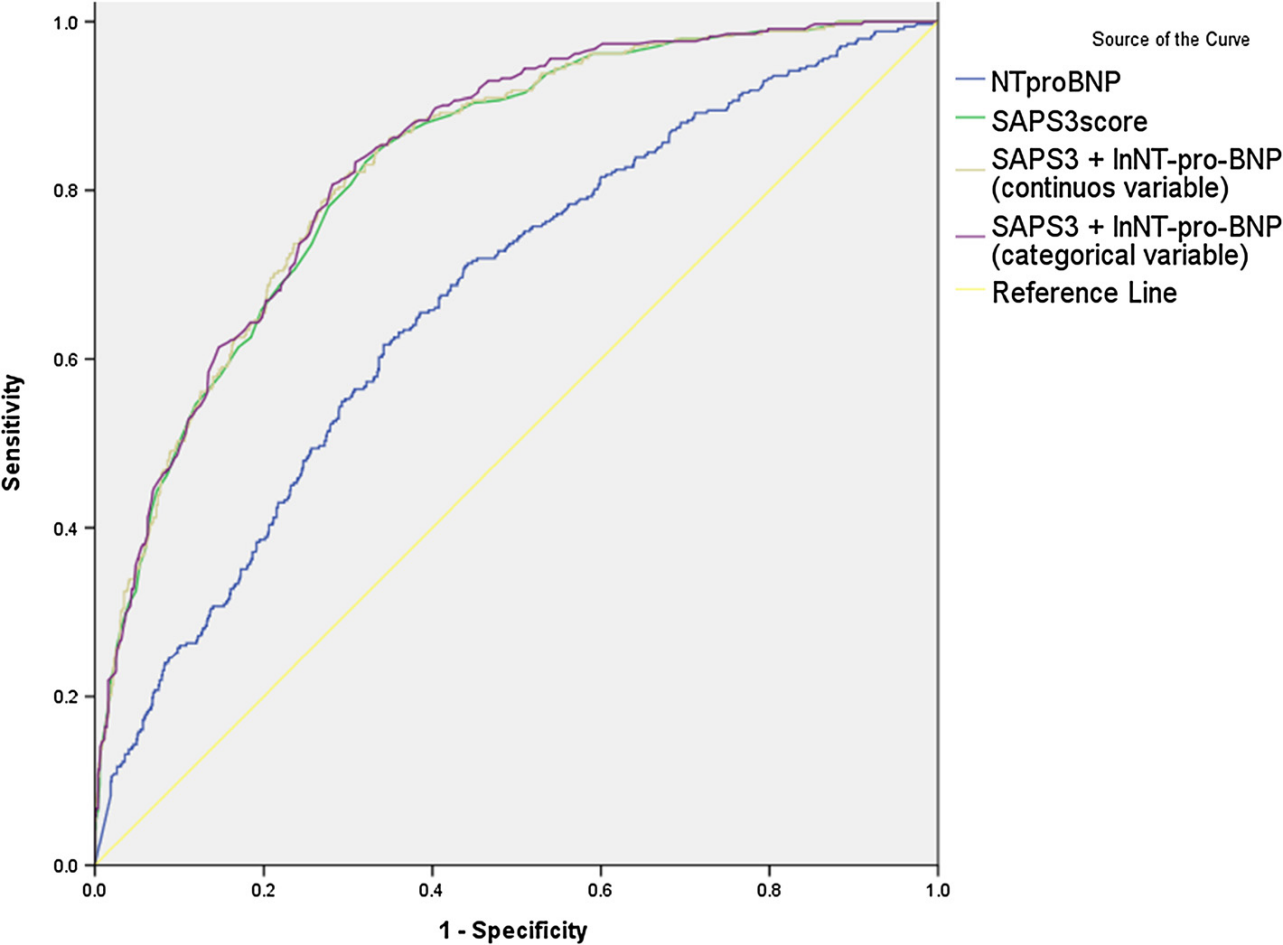
**Receiver operating characteristic (ROC) curve analysis for hospital mortality.** The area under the ROC curve was 0.67 for the NT-pro-BNP level and 0.83 for the SAPS 3 score. Combine of NT-pro-BNP level and SAPS 3 score resulted in little additional power.

ROC curve analysis was used to identify the NT-pro-BNP level and SAPS 3 score on admission that best predicted hospital mortality. An NT-pro-BNP concentration of 514.8 pg/mL had a sensitivity of 62% and specificity of 66% for predicting hospital mortality. Positive predictive value was 0.37 and negative predictive value was 0.84. The area under the ROC curve was 0.67 (95% confidence interval [CI], 0.64–0.70). A SAPS 3 score of 60.5 had a sensitivity of 81% and specificity of 70% for predicting hospital mortality. The area under the ROC curve was 0.83 (95% CI, 0.81–0.85). AUC increment by adding NT-pro-BNP is minimal and likely no different to SAPS 3 alone (Table 4, Figure 4).

**Table 4:** The area under the ROC curve in prediction of hospital mortality

	**AUC**	**95% CI**	***p*-value**
NT-pro-BNP	0.671	0.639-0.704	<0.001
SAPS3	0.828	0.805-0.852	<0.001
SAPS3 + ln_NT-pro-BNP_ (continuous variable)	0.831	0.808-0.855	<0.001
SAPS3 + ln_NT-pro-BNP_ (categorical variable)	0.835	0.812-0.858	<0.001

ROC = Receiver operating characteristic; AUC = area under the curve; CI = confidence interval; SAPS 3 = Simplified Acute Physiology Score (SAPS) 3.

Natural-log-transformed (ln) NT-pro-BNP was used as the continuous variable or divided by the quartile and used as the categorical variable.

**Figure 5:**
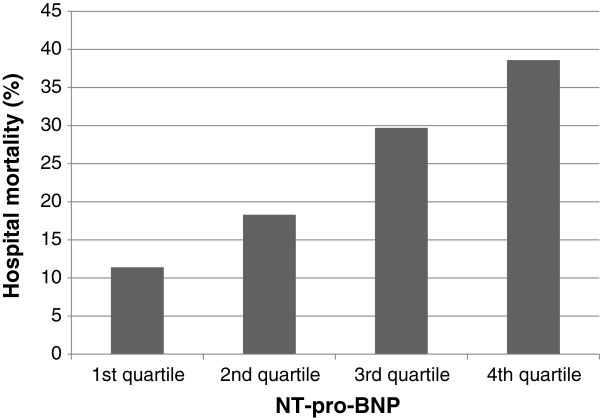
**Hospital mortality according to NT-pro-BNP level.** Mortality increased with the quartile of the NT-pro-BNP level.

Almost all clinical characteristics differed significantly according to NT-pro-BNP level (Table 5). The percentage of ischemic heart disease, congestive heart failure, and chronic renal failure were increased according to NT-pro-BNP level quartile. Among the reasons for ICU admission, the rates of cardiovascular and renal complications were increased according to the NT-pro-BNP level quartile. SAPS 3 score, SOFA score, ICU day, and ICU mortality also increased according to NT-pro-BNP level quartile. Hospital mortality also differed significantly according to NT-pro-BNP level (11.4% in the first quartile, 18.3% in the second quartile, 29.7% in the third quartile, and 38.6% in the fourth quartile; *p* < 0.001; Figure 5).

**Table 5:** Comparison of clinical characteristics according to the quartile of NT-pro-BNP level

**NT-pro-BNP (pg/mL)**	**< 104**	**104–341**	**341–1,637**	**> 1,637**	***p*-value**
Number of patients	360	360	360	360	
Age, years	59 (49–69)	63 (52–72)	67 (55–76)	68 (56–75)	< 0.001
Male	250 (69.4)	223 (61.9)	213 (59.2)	220 (61.1)	0.024
Comorbidities					
Cirrhosis	47 (13.1)	62 (17.2)	29 (8.1)	19 (5.3)	< 0.001
Hypertension	118 (32.8)	120 (33.3)	142 (39.4)	164 (45.6)	0.001
IHD	18 (5.0)	29 (8.1)	49 (13.6)	64 (17.8)	< 0.001
CHF	5 (1.4)	18 (5.0)	37 (10.3)	48 (13.3)	< 0.001
DM	75 (20.8)	71 (19.7)	94 (26.1)	123 (34.2)	< 0.001
CRF	8 (2.2)	16 (4.4)	35 (9.7)	85 (23.6)	< 0.001
Cancer	130 (36.1)	164 (45.6)	144 (40.0)	106 (29.4)	< 0.001
Status at ICU admission					
Severe sepsis or septic shock	42 (11.7)	64 (17.8)	75 (20.8)	128 (35.6)	< 0.001
ALI or ARDS	18 (5.0)	27 (7.5)	28 (7.8)	50 (13.9)	< 0.001
Admission category					
Medical	171 (47.5)	159 (44.2)	212 (58.9)	293 (81.4)	< 0.001
Surgical	189 (52.5)	201 (55.8)	148 (41.1)	67 (18.6)	< 0.001
Reason for ICU admission					
Basic & observational^**^	166 (46.1)	174 (48.3)	156 (43.3)	87 (24.2)	< 0.001
Cardiovascular	40 (11.1)	47 (13.1)	58 (16.1)	119 (33.1)	< 0.001
Digestive	22 (6.1)	14 (3.9)	15 (4.2)	11 (3.1)	0.236
Hepatic failure	29 (8.1)	39 (10.8)	14 (3.9)	4 (1.1)	< 0.001
Neurologic	10 (2.8)	5 (1.4)	9 (2.5)	11 (3.1)	0.519
Renal	1 (0.3)	2 (0.6)	3 (0.8)	15 (4.2)	< 0.001
Respiratory	61 (19.6)	66 (18.3)	90 (25.0)	93 (25.8)	0.004
SAPS 3 score	50 (39–61)	55 (47–65)	58 (49–68)	68 (57–78)	< 0.001
SOFA score	5 (2–9)	6 (3–10)	6 (3–11)	10 (6–13)	< 0.001
ICU day	5 (3–12)	6 (4–16)	7 (4–17)	9 (4–20)	< 0.001
ICU mortality	28 (7.8)	55 (15.3)	76 (21.1)	113 (31.4)	< 0.001
Hospital day	16 (9–31)	17 (11–35)	17 (11–37)	19 (10–38)	0.106
Hospital mortality	41 (11.4)	66 (18.3)	107 (29.7)	139 (38.6)	< 0.001

^**^Basic and observational intensive care was defined as the patient being in the ICU for surveillance, simple weaning from a ventilator after surgery, routine post-surgery care, or needing complex nursing care or monitoring drug intoxication without organ dysfunction.

IHD = ischemic heart disease; CHF = congestive heart failure; CPF = chronic pulmonary disease; DM = diabetes mellitus; CRF = chronic renal failure; ICU = intensive care unit; ALI = acute lung injury; ARDS = acute respiratory distress syndrome.

Logistic regression was performed to determine whether the NT-pro-BNP level has additional power to predict hospital mortality in combination with the SAPS 3 score. The odds ratio of the SAPS 3 score alone was 1.10. When combined with NT-pro-BNP, the odds ratio of the SAPS 3 score was 1.09 (Tables 6 and 7). There was little change in the odds ratio and 95% CI of SAPS 3 when combined with NT-pro-BNP.

**Table 6:** Logistic regression for prediction of hospital mortality (SAPS 3 alone)

	**Odds ratio**	**95% CI**	***p*-value**
SAPS 3 score	1.10	1.08-1.10	< 0.001

Goodness of fit (Hosmer–Lemeshow) chi-squared *p*-value = 0.119. CI = confidence interval; SAPS 3 = Simplified Acute Physiology Score (SAPS) 3.

**Table 7:** Logistic regression for prediction of hospital mortality (SAPS 3 + NT-pro-BNP)

	**Odds ratio**	**95% CI**	***p*-value**
SAPS 3 score	1.09	1.08-1.11	< 0.001
ln_NT-pro-BNP_	1.13	1.05-1.21	0.001

Goodness of fit (Hosmer–Lemeshow) chi-squared *p*-value = 0.146. Natural-log-transformed (ln) NT-pro-BNP was used as the continuous variable.

CI = confidence interval; SAPS 3 = Simplified Acute Physiology Score (SAPS) 3.

## Discussion

In the present study, we showed that the serum level of NT-pro-BNP on admission has weak prognostic power and little additive prognostic power when combined with SAPS 3 score. Although previous studies [7-10] have shown that the NT-pro-BNP level can be a prognostic factor in critically ill patients, the present study represents the largest scale study.

In the present study, the serum NT-pro-BNP level on admission was significantly higher in ICU nonsurvivors than in survivors. Moreover, the mortality progressively increased with increasing levels of NT-pro-BNP. Our findings are compatible with previous reports. All four studies [7-10] consistently showed similar results.

Compared with previous studies, the prognostic power of NT-pro-BNP was similar or somewhat weaker. Almog *et al*. [7] showed that the area under the ROC curve of NT-pro-BNP levels for prediction of mortality was 0.75 (95% CI, 0.62–0.88). Meyer *et al*. [8] showed that the area under the ROC curve was 0.70 (95% CI, 0.64–0.77). In the present study, the area under the ROC curve was 0.67 (95% CI, 0.64–0.70). The reason for the lower prognostic power in the present study is unknown. To address this issue, additional validation of the prognostic power of NT-pro-BNP is needed. One possible explanation is that the population enrolled in the two previous studies may have been biased. Thus, the prognostic power of NT-pro-BNP might be overestimated. In the study by Almog and colleagues [7], the number of patients analyzed was only 78, and all were admitted to the medical ICU in a single center. In the study by Meyer and colleagues [8], 289 patients in only a single center were evaluated. Although Meyer *et al*. [8] included the entire spectrum of medical patients with a critical illness, many had cardiac disease because the ICU in that study was in the Department of Cardiology. In those patients, the NT-pro-BNP level was more likely to have prognostic power. Compared with the previous two studies, a much larger number of number of patients were enrolled in the present study (n = 1440), and they were admitted not only to the medical ICU but also to the surgical ICU at multiple centers. Single measurement of the NT-pro-BNP level on admission can be an attractive prognostic factor if it is sufficiently powerful because other scoring systems, such as the SAPS 3, are somewhat complex and time consuming. Unfortunately, our data suggest that the NT-pro-BNP level alone is not a powerful prognostic factor in patients admitted to the ICU.

Along with its weak power for prediction of mortality, our data suggest that the NT-pro-BNP level plays little adjunct role when combined with SAPS 3 score. This result is not compatible with previous reports. Meyer *et al*. [8] showed that the SAPS 2 score and the NT-pro-BNP level were independently associated with hospital survival in a logistic regression model. Kotanidou *et al*. [9] reported that the Acute Physiology and Chronic Health Evaluation (APACHE) II score and NT-pro-BNP level were independent predictors of mortality in multiple logistic regression analysis. The reason for the NT-pro-BNP level having little adjunct role in this study when combined with SAPS 3 is unknown. One possible explanation is that, in the present study, the NT-pro-BNP level was well correlated with many clinical parameters that are components of SAPS 3. Thus, prognosis may be already predicted enough by only SAPS 3 score.

In the present study, when combined with the SAPS 3 score, the AUC was increased little. As shown in Tables 3, many clinical parameters and characteristics were overlapped by the SAPS 3 score and NT-pro-BNP level. Thus, when analyzed using the combination of the SAPS 3 and NT-pro-BNP level, the power of NT-pro-BNP was offset. For example, renal complication is the reason whose level of NT-pro-BNP is highest among the reason of ICU admission (Figure 3). This suggests that the prognostic power of NT-pro-BNP is due partly to the poor prognosis of renal failure patients. However, blood urea nitrogen, creatinine, pH, HCO_3_^-^, and urine output are components of the SAPS 3 score. The NT-pro-BNP level was also well correlated with these parameters, which are important prognostic factors in patients with renal failure. The poor prognosis of renal failure is already sufficiently predicted by the SAPS3 score. Thus, NT-pro-BNP had only a limited effect upon combination with the SAPS 3 score.

Conversely to usual biomarkers, severity score have been proven to be poorly calibrated to predict individual mortality. This may be because of differences in the patient case-mix or changing medical practice over time [11]. For example, SAPS 3 score showed mixed result in the external validation studies [12-17]. In this study, this phenomenon was also observed. Although SAPS 3 score was independent predictor for mortality, the area under the ROC curve was only 0.83. For NT-pro-BNP, there has been also variability for the power to predict mortality in previous studies. Thus, further validation study for NT-pro-BNP is mandatory.

The limitation of this study is potential selection bias. In this study, there were no definite criteria for the measurement of NT-pro-BNP. Basically, this study was multicenter observational study. Thus, in some centers, NT-pro-BNP was measured routinely for all patients, while in other centers, NT-pro-BNP was measured by clinician’s preference. Thus, selection bias for enrollment of patients may exist. However, the level of NT-pro-BNP was variable, which suggests that patients who were unlikely to have cardiac disease were also enrolled. Baseline characteristics also showed that the percentage of ischemic heart disease or heart failure was not high (11.1 and 7.5%, respectively), which supports selection bias for patients with cardiac disease was unlikely to occur.

The present study is valuable in that it was of a multicenter, prospective design. In the present study, multiple ICU settings (medical or surgical or multidisciplinary) in multiple centers were analyzed. Thus, the enrolled population may be representative of real-world ICU patients. To be a good prognostic marker, NT-pro-BNP should be validated in a large-scale multicenter study. Our results provide valuable information regarding the limited role of NT-pro-BNP in general ICU patients. Further investigation of the role of NT-pro-BNP in critically ill patients with various clinical characteristics is needed.

## Conclusions

In conclusion, the NT-pro-BNP level was more elevated in nonsurvivors in a multicenter cohort of critically ill patients. However, there was little additional prognostic power when adding NT-pro-BNP to SAPS 3 score. Further investigation of NT-pro-BNP as a prognostic factor in patients admitted to the ICU is needed.

## Competing interests

The authors declare that they have no competing interests.

## Authors’ contributions

RCK, LSY, KSH, CWI, LYJ, CGR, KJH, KJY, LJ, PS, KHC, LJH, LJH, PJ, KY, SGY, and KSC participated in the design of the study. RCK, LSY, KSH, CWI, LYJ, CGR, KJH, KJY, LJ, PS, KHC, LJH, LJH, PJ, KY, SGY, and KSC participated in the collection of data. RCK, LSY, SGY and KSC performed the statistical analysis. RCK and KSC wrote the manuscript. All authors read and approved the final manuscript.

## Pre-publication history

The pre-publication history for this paper can be accessed here:

http://www.biomedcentral.com/1471-2253/14/16/prepub
